# Sociodemographic factors and delays in the diagnosis of six cancers: analysis of data from the ‘*National Survey of NHS Patients: Cancer*’

**DOI:** 10.1038/sj.bjc.6602623

**Published:** 2005-05-17

**Authors:** R D Neal, V L Allgar

**Affiliations:** 1 Department of General Practice, North Wales Clinical School, Wales College of Medicine, Cardiff University, Wrexham Technology Park, Wrexham LL13 7YP, UK; 2 Centre for Research in Primary Care, University of Leeds, 71-75 Clarendon Road, Leeds LS2 9PL, UK

**Keywords:** delay, diagnosis, pre-hospital, secondary care, referral, sociodemographic

## Abstract

This paper aims to explore the relationship between sociodemographic factors and the components of diagnostic delay (total, patient and primary care, referral, secondary care) for these six cancers (breast, colorectal, lung, ovarian, prostate, or non-Hodgkin's lymphoma). Secondary analysis of patient-reported data from the ‘National Survey of NHS patients: Cancer’ was undertaken (65 192 patients). Data were analysed using univariate analysis and Generalised Linear Modelling. With regard to total delay, the findings from the GLM showed that for colorectal cancer, the significant factors were marital status and age, for lung and ovarian cancer none of the factors were significant, for prostate cancer the only significant factor was social class, for non-Hodgkin's lymphoma the only significant factor was age, and for breast cancer the significant factors were marital status and ethnic group. Where associations between any of the component delays were found, the direction of the association was always in the same direction (female subjects had longer delays than male subjects, younger people had longer delays than older people, single and separated/divorced people had longer delays than married people, lower social class groups had longer delays than higher social class groups, and Black and south Asian people had longer delays than white people). These findings should influence the design of interventions aimed at reducing diagnostic delays with the aim of improving morbidity, mortality, and psychological outcomes through earlier stage diagnosis.

The accompanying paper (Allgar and Neal, submitted) summarises the important literature describing diagnostic delays in six cancers (breast, colorectal, lung, ovarian, prostate, or non-Hodgkin's lymphoma (NHL)), and discusses the importance of diagnostic delay. This paper explores the relationship between sociodemographic factors and the components of diagnostic delay (total, patient and primary care, referral, secondary care) for these six cancers, about which there is a small body of literature in breast and colorectal cancer, but not in prostate, ovarian, or lung cancer or NHL.

For breast cancer, there are conflicting findings with respect to age. No associations have been reported with patient delays (Nosarti *et al*, 2000; Meechan *et al*, 2002) or physician delays (Tartter *et al*, 1999). Longer delays have been associated with older age (Arndt *et al*, 2002), but faster times to treatment have also been associated with increasing age (Robertson *et al*, 2004). Other positive findings from the literature include: African-American women having longer delays than white women (Gwyn *et al*, 2004), and unmarried women having longer patient delays than married women (Thongsuksai *et al*, 2000). Other negative findings include: no other socioeconomic factors being important in patient delays (Thongsuksai *et al*, 2000); no sociodemographic factors being important in patient delay (Meechan *et al*, 2002); and socioeconomic status and ethnicity not being contributory to referral delays (Nosarti *et al*, 2000). Similarly, there are conflicting findings from the colorectal literature, although this is more limited. One paper has reported faster time to treatment in patients aged 50–74 years (Robertson *et al*, 2004), another has reported that age and gender were not associated with differences in delays (Gonzalez-Hermoso *et al*, 2004); and another that marital status is one of several multifactorial reasons for delay (Langenbach *et al*, 2003).

This paper aims to explore the relationship between sociodemographic factors and the components of diagnostic delay (total, patient and primary care, referral, secondary care) for these six cancers, using patient-reported data from the National Survey of NHS patients: Cancer (DoH, 2002). If associations exist between sociodemographic factors and diagnostic delays, this should influence the design of interventions aimed at reducing diagnostic delays with the aim of improving morbidity, mortality, and psychological outcomes through earlier stage diagnosis.

## MATERIALS AND METHODS

### Data source and calculating delays

The accompanying paper contains details regarding from The National Survey of NHS Patients: Cancer (DoH, 2002) and our analysis of data to calculate delays therefrom (Allgar and Neal, submitted). In summary, the survey collected data from 65 192 patients with one of six types of cancer (female breast, colorectal, prostate, NHL, lung, and ovarian) from NHS Trusts in England. Various components of delays (patient and primary care delays, referral delays, secondary delays, and total delays) were calculated from answers to questions about their cancer journey. Owing to different diagnostic pathways and ways in which the survey questions were asked, delays were calculated differently for patients who reported seeing their GP prior to diagnosis than for those that did not (diagnosed by screening, direct hospital admission, or interspecialty referral).

### Sociodemographic factors

The survey collected demographic data relating to age, sex, social class, marital status, and ethnic group. Age was calculated by subtracting date of birth from the date that the patient first saw a hospital doctor for their cancer, and was then categorised into seven groups (<25, 25–34, 35–44, 45–54, 55–64, 65–74, and 75+ years) for the univariate comparisons. Marital status was classified as ‘married/living with partner’, ‘divorced/separated’, ‘widowed’, or ‘single’. Social class was derived from occupation using the Registrar General categorisation ‘professional’, ‘managerial/technical’, ‘skilled nonmanual’, ‘skilled manual’, ‘partly skilled’, ‘unskilled’, ‘armed forces’, and ‘never worked’. Ethnic group was further categorised to ensure there were adequate numbers in each category: White; Black (Black-Caribbean, Black-African, and Black–other); South Asian (Indian, Pakistani, and Bangladeshi); and Other (Chinese and ‘other’). There was some missing data for the sociodemographic factors, which accounts for the individual category totals in Table 1 sometimes not equalling the base number for each group.

### Statistics

Initially *T*-tests and ANOVA were used for each cancer group to compare mean delay between the categorical sociodemographic factors: age categories, sex, marital status, ethnic group, and social class. However, the univariate analysis makes no allowance for confounding factors (e.g. age, sex, marital status, ethnic group, and social class). Generalised Linear Modelling (GLM) was therefore used to investigate which were the most important factors associated with variation in delay, while controlling for the potentially confounding factors. This was undertaken for each cancer group (age was included as a continuous variable, rather than using the age categories). Generalised Linear Modelling provides regression analysis and analysis of variance for one dependent variable (components of delay) by one or more factors. It allows testing of the null hypotheses about the effects of other factors on the means of various groupings of a single dependent variable. For regression analysis, the independent (predictor) variables are specified as covariates (age, sex, marital status, ethnic group, and social class). A *P*-value of <0.05 was used to indicate statistical significance. All analyses were performed on SPSS (Version 11).

## RESULTS

The main results are presented in Tables 1,2 and 3. Table 1 shows the mean delay and standard deviation for each of the component delays, for each of the cancers, and for each of the sociodemographic factors. Table 2 shows the results of the univariate analysis, and Table 3 the results of the GLM. The direction of the trends where there were differences between groups were all in the same direction. These were as follows: sex – female subjects had longer delays than males; age – younger people had longer delays than older people; marital status – single and separated/divorced people had longer delays than married people; social class – lower social class groups had longer delays than higher social class groups; and ethnic group – Black and south Asian people had longer delays than white people.

### Total delay

#### Individual sociodemographic factors

There was a significant difference in delay and age group for colorectal, lung, NHL, and breast. There was a significant difference in delay and marital status for colorectal, lung, and breast cancer. There was a significant difference in delay and ethnic group for breast cancer.

#### Generalised Linear Modelling

For colorectal cancer, the significant factors were marital status and age. For lung cancer, none of the factors were significant. For ovarian cancer, none of the factors were significant. For prostate cancer, the only significant factor was social class. For NHL, the only significant factor was age. For breast cancer, the significant factors were marital status and ethnic group.

### Pre-hospital delay

#### Individual sociodemographic factors

There was a significant difference in delay and age group for lung, NHL, and breast. There was a significant difference in delay and marital status for colorectal and breast cancer. There was a significant difference between delay and ethnic group for breast cancer.

#### Generalised Linear Modelling

For colorectal cancer, the only significant factor was marital status. For lung cancer, the only significant factor was age. For ovarian cancer, none of the factors were significant. For prostate cancer, none of the factors were significant. For NHL, the only significant factor was age. For breast cancer, the significant factors were marital status and ethnic group.

### Referral delay

#### Individual sociodemographic factors

There was a significant difference in delay and age group for all six cancers. There was a significant difference between male and female subjects for colorectal and NHL. There was a significant difference in delay and marital status for colorectal and breast cancer. There was a significant difference in delay and social class for colorectal cancer. There was a significant difference in delay and ethnic group for colorectal, prostate, and breast cancer.

#### Generalised Linear Modelling

For colorectal cancer, the significant factors were sex, ethnic group, and age. For lung cancer, the only significant factor was age. For ovarian cancer, none of the factors were significant. For prostate cancer, the only significant factor was age. For NHL, the only significant factor was age. For breast cancer, the significant factors were marital status and age.

### Secondary care delay

#### Individual sociodemographic factors

There was a significant difference in delay and age group for colorectal, lung, prostate, NHL, and breast cancer. There was a significant difference between sex and delay for colorectal and lung. There was a significant difference in delay and marital status for colorectal, prostate, NHL, and breast cancer. There was a significant difference in delay and social class for colorectal, ovarian, prostate, and breast cancer. There was a significant difference in delay and ethnic group for lung cancer.

#### Generalised Linear Modelling

For colorectal cancer, the significant factors were sex, marital status, social class, and age. For lung cancer, the only significant factor was ethnic group and age. For ovarian cancer, the only significant factor was social class. For prostate cancer, the significant factors were marital status, social class, and age. For NHL, the significant factors were sex, marital status, and age. For breast cancer, the significant factors were marital status, social class, and age.

## DISCUSSION

The main findings of this study show significant associations between some of the sociodemographic variables and some of component delays in these six cancers. These findings have significant implications for further research and for policy development.

The GLM showed that the significant factors varied by cancer type. Looking at total delay, for colorectal, age, and marital status were the key factors in explaining the variation in delays; this strengthens the limited evidence base to date (Langenbach *et al*, 2003; Gonzalez-Hermoso *et al*, 2004; Robertson *et al*, 2004). For lung and ovarian cancer, none of the factors stood out as being important. For prostate, social class was an important factor. For NHL, age was an important factor. For breast cancer, marital status and ethnic group were important factors, again strengthening the current evidence base (Tartter *et al*, 1999; Nosarti *et al*, 2000; Thongsuksai *et al*, 2000; Arndt *et al*, 2002; Meechan *et al*, 2002; Gwyn *et al*, 2004; Robertson *et al*, 2004). The trends in the associations using both statistical approaches were all in the same direction for each of the six cancers. The findings for each of the component delays demonstrate the importance of the sociodemographic factors on that stage in the cancer diagnostic journey. The findings for pre-hospital delay most closely mirror the findings for total delays since this is the part of the process where the majority of the delay occurs (Allgar and Neal, submitted). The small, but statistically significant findings for referral and secondary care delay may be of less clinical significance.

Where gender differences existed, female subjects had longer delays than male subjects; this was an unexpected finding, and the reasons for it are unclear and warrant further investigation. Where age differences existed, younger people had longer delays than older people. This may be because cancer is rarer in younger people, so is more likely to go unnoticed by both patients and their health professionals. Where marital status differences existed, single and separated/divorced people had longer delays than married people. The presence of a partner may facilitate earlier diagnosis by noticing symptoms, discussing the meaning of symptoms, and encouraging their presentation to a health professional. Where social class differences existed, lower social class groups had longer delays than higher social class groups. This may be as a result of lower levels of knowledge regarding significant symptoms, and as a result of poorer access to services. Where ethnic group differences existed, Black and south Asian people had longer delays than white people. This may be a result of primary care being slow to provide accessible care appropriate to the needs of minority ethnic populations, and the health care needs of South Asian patients being either ignored or, if they are recognised, subject to various stereotypes and myths (Atkin, 2004).

### Strengths and limitations

The strengths and limitations of the data analysed in this paper are discussed in full in the accompanying paper (Allgar and Neal, submitted). In summary, the analysis was based on a large, high-quality data set. Limitations of the data set include the number of patients who had died prior to receiving the survey, recall bias due to time from diagnosis, and lack of data relating to diagnostic stage and comorbidity. In our analysis, various assumptions concerning the data had to be made in the calculation of delays, and, despite the large numbers overall, the numbers of patients in younger age groups for some cancers was small. As a result, our findings must be interpreted with some caution, and may need replicating with other data.

### Implications for further research and policy development

Interventions intended to reduce delay (e.g. the urgent suspected cancer referral guidance) need to be appropriate for the population. Research is needed to develop and evaluate interventions aimed at specific groups in order to reduce diagnostic delays with the aim of improving morbidity, mortality, and psychological outcomes through earlier stage diagnosis. The findings of this work will inform the process of who those interventions should be aimed at, and at what stage of the cancer diagnostic journey they are likely to impact upon.

## Figures and Tables

**Table 1:** Mean delays (s.d.) for each component of delay for all cancers for each sociodemographic variable

	**Total delays (days)**						**Patient and primary care delays (days)**						**Referral delays (days)**						**Secondary care delays (days)**					
	**CRC**	**L**	**O**	**P**	**NHL**	**B**	**CRC**	**L**	**O**	**P**	**NHL**	**B**	**CRC**	**L**	**O**	**P**	**NH**	**B**	**CRC**	**L**	**O**	**P**	**NHL**	**B**
***n***	**11 385**	**2669**	**2216**	**5840**	**3537**	**19 760**	**13 174**	**3260**	**2673**	**7759**	**4650**	**22 494**	**12 527**	**2950**	**2453**	**7877**	**4242**	**17 402**	**13 244**	**3199**	**2474**	**7671**	**4073**	**21 938**
*Sex*																								
Male	120 (451)	87 (258)	n/a	148 (494)	108 (317)	n/a	111 (444)	77 (254)	n/a	142 (506)	104 (389)	n/a	41 (53)	32 (47)	n/a	50 (57)	35 (51)	n/a	11 (24)	11 (22)	n/a	11 (25)	13 (24)	n/a
Female	133 (307)	91 (208)	90 (320)	n/a	97 (167)	55 (242)	123 (399)	82 (281)	83 (307)	n/a	95 (289)	52 (250)	45 (56)	35 (51)	35 (51)	n/a	40 (55)	21 (31)	13 (26)	13 (24)	9 (20)	n/a	14 (26)	5 (14)

*Age (years)*																								
<25	94 (86)	127 (130)	121 (390)	—	63 (74)	47 (38)	75 (95)	103 (88)	108 (356)	—	108 (524)	47 (85)	49 (70)	36 (51)	12 (19)	9 (4)	39 (55)	26 (35)	22 (40)	7 (0)	2 (5)	—	10 (15)	15 (15)
25–34	148 (205)	151 (243)	84 (140)	—	134 (240)	75 (172)	123 (185)	117 (202)	93 (164)	31 (61)	193 (710)	57 (157)	62 (71)	62 (63)	41 (54)	30 (54)	39 (58)	31 (41)	20 (36)	18 (32)	9 (20)	—	19 (30)	13 (23)
35–44	155 (207)	190 (450)	80 (134)	197 (430)	104 (144)	60 (159)	135 (203)	148 (393)	77 (130)	104 (283)	117 (495)	54 (157)	51 (63)	48 (65)	43 (58)	28 (56)	38 (55)	24 (34)	17 (27)	21 (37)	11 (20)	23 (24)	17 (28)	8 (17)
45–54	160 (369)	95 (247)	98 (184)	221 (641)	128 (295)	52 (165)	142 (365)	107 (444)	88 (171)	208 (644)	108 (277)	47 (175)	51 (61)	40 (62)	35 (53)	51 (53)	43 (60)	22 (32)	15 (27)	13 (22)	8 (20)	22 (34)	18 (30)	7 (15)
55–64	137 (513)	79 (181)	96 (512)	162 (358)	101 (268)	45 (192)	127 (497)	67 (175)	84 (472)	146 (372)	90 (295)	43 (208)	44 (56)	32 (49)	36 (52)	52 (55)	33 (48)	19 (28)	12 (24)	13 (24)	9 (21)	21 (32)	14 (25)	5 (12)
65–74	121 (322)	94 (293)	72 (131)	146 (536)	93 (233)	60 (358)	112 (341)	82 (283)	67 (149)	144 (574)	87 (240)	61 (367)	41 (53)	31 (44)	32 (49)	49 (55)	36 (51)	17 (24)	13 (26)	11 (22)	8 (20)	13 (27)	12 (24)	4 (10)
75+	105 (384)	74 (137)	114 (257)	139 (496)	85 (290)	68 (338)	102 (492)	64 (134)	117 (348)	131 (474)	77 (272)	70 (343)	37 (49)	31 (42)	28 (33)	45 (55)	33 (47)	18 (26)	10 (23)	10 (22)	9 (22)	6 (17)	8 (18)	2 (8)

*Marital status*																								
Married/living with partner	123 (272)	85 (211)	91 (371)	149 (465)	106 (255)	51 (213)	110 (273)	76 (251)	82 (349)	143 (495)	102 (359)	47 (220)	44 (55)	33 (49)	34 (50)	51 (57)	36 (52)	21 (31)	13 (26)	12 (23)	9 (21)	13 (26)	14 (26)	6 (13)
Divorced/separated	140 (357)	139 (476)	89 (128)	127 (237)	113 (185)	59 (221)	171 (802)	121 (439)	92 (148)	132 (327)	96 (196)	56 (233)	47 (59)	36 (54)	40 (63)	48 (60)	40 (58)	23 (30)	11 (23)	13 (27)	7 (14)	10 (26)	16 (30)	6 (16)
Widowed	111 (285)	90 (240)	85 (169)	144 (674)	91 (339)	67 (329)	104 (303)	82 (263)	80 (196)	136 (605)	88 (318)	67 (332)	40 (53)	31 (44)	35 (50)	47 (59)	38 (53)	20 (31)	10 (22)	10 (21)	9 (21)	6 (18)	8 (19)	3 (10)
Single	196 (1215)	66 (106)	97 (170)	185 (629)	89 (149)	69 (291)	173 (1130)	54 (94)	93 (179)	165 (570)	104 (375)	70 (308)	39 (51)	35 (50)	38 (59)	51 (61)	42 (56)	22 (30)	11 (23)	10 (23)	9 (20)	9 (21)	14 (23)	7 (17)

*Social class*																								
Professional	109 (159)	91 (124)	157 (283)	133 (227)	85 (159)	56 (169)	96 (161)	73 (105)	116 (230)	118 (234)	69 (172)	48 (158)	37 (49)	30 (48)	41 (56)	48 (53)	30 (48)	18 (22)	14 (27)	17 (31)	22 (37)	15 (30)	13 (22)	6 (14)
Managerial/technical	124 (258)	85 (141)	107 (387)	147 (406)	99 (204)	55 (215)	117 (286)	74 (134)	97 (356)	144 (509)	91 (208)	51 (217)	42 (52)	36 (54)	37 (53)	49 (55)	38 (55)	21 (31)	12 (25)	11 (22)	9 (21)	13 (27)	14 (25)	7 (16)
Skilled nonmanual	146 (710)	74 (99)	81 (124)	148 (526)	95 (156)	50 (188)	138 (778)	59 (91)	76 (172)	141 (505)	104 (322)	48 (212)	44 (53)	36 (51)	36 (52)	49 (56)	36 (50)	21 (31)	14 (28)	14 (25)	9 (20)	12 (25)	15 (28)	6 (13)
Skilled manual	125 (329)	95 (332)	101 (234)	151 (592)	132 (413)	62 (261)	117 (332)	87 (330)	90 (219)	150 (574)	120 (429)	58 (266)	43 (56)	33 (47)	39 (53)	53 (59)	38 (53)	21 (33)	11 (24)	12 (24)	9 (20)	11 (23)	15 (27)	5 (13)
Partly skilled	130 (322)	91 (227)	70 (111)	144 (308)	103 (230)	51 (171)	118 (352)	83 (238)	71 (130)	134 (311)	104 (461)	47 (184)	46 (59)	33 (47)	33 (52)	51 (60)	38 (56)	22 (33)	12 (24)	11 (22)	9 (21)	11 (25)	13 (26)	6 (13)
Unskilled	103 (144)	101 (35)	66 (83)	295 (1126)	97 (140)	60 (227)	90 (140)	122 (596)	59 (83)	247 (988)	85 (153)	53 (215)	42 (53)	30 (51)	30 (37)	59 (65)	45 (62)	19 (25)	12 (24)	13 (24)	10 (24)	12 (25)	12 (25)	4 (11)
Armed forces	96 (140)	55 (35)	—	342 (720)	124 (163)	62 (37)	119 (140)	45 (20)	—	226 (494)	89 (156)	55 (33)	74 (98)	130 (121)	—	57 (54)	35 (39)	17 (21)	11 (11)	9 (11)	—	6 (15)	16 (24)	7 (9)
Never worked	126 (472)	106 (356)	172 (961)	111 (161)	85 (144)	59 (251)	108 (429)	85 (321)	150 (887)	96 (160)	141 (678)	66 (318)	44 (57)	37 (49)	36 (61)	48 (66)	36 (56)	20 (31)	10 (24)	13 (24)	5 (14)	9 (26)	9 (16)	4 (11)

*Ethnic group*																								
White	125 (397)	88 (242)	90 (322)	149 (500)	102 (258)	54 (238)	116 (427)	79 (268)	83 (309)	142 (510)	100 (349)	51 (245)	42 (54)	33 (49)	35 (51)	50 (57)	37 (53)	21 (30)	12 (25)	11 (23)	9 (21)	11 (25)	13 (25)	5 (13)
Black	217 (515)	102 (63)	34 (43)	113 (170)	150 (328)	68 (118)	188 (468)	100 (142)	39 (52)	141 (416)	122 (307)	66 (121)	67 (77)	42 (34)	17 (21)	58 (60)	35 (48)	26 (34)	11 (24)	27 (37)	1 (3)	13 (27)	14 (25)	8 (17)
South Asian	140 (191)	77 (130)	165 (363)	80 (120)	101 (178)	103 (475)	177 (492)	59 (105)	125 (282)	72 (113)	96 (173)	107 (529)	59 (67)	43 (49)	32 (44)	81 (87)	38 (47)	24 (35)	9 (20)	11 (32)	2 (3)	10 (30)	9 (20)	6 (13)
Other	140 (196)	86 (96)	83 (95)	86 (108)	118 (264)	91 (282)	122 (177)	85 (141)	70 (88)	71 (94)	99 (243)	79 (263)	66 (69)	43 (66)	57 (71)	53 (43)	38 (63)	29 (39)	15 (28)	33 (37)	9 (20)	7 (17)	22 (38)	5 (13)

CRC=colorectal cancer, L=lung cancer, O=ovarian cancer, P=prostate cancer, NHL=non-Hodgkin lymphoma, B=breast cancer n/a=not applicable.

**Table 2:** Summary of significant findings from univariate analysis

	**Colorectal**	**Lung**	**Ovarian**	**Prostate**	**NHL**	**Breast**
*Total delay*						
Sex	NS	NS	n/a	n/a	NS	n/a
Age	F(6)=3.640, *P*=0.001	F(6)=2.450, *P*=0.023	NS	NS	F(6)=2.481, *P*=0.021	F(6)=3.754, *P*=0.001
Marital status	F(3)=7.922, *P*<0.001	F(3)=3.074, *P*=0.027	NS	NS	NS	F(3)=5.283, *P*=0.001
Social class	NS	NS	NS	NS	NS	
Ethnic group	NS	NS	NS	NS	NS	F(3)=3.501, *P*=0.015

*Pre-hospital delay*						
Sex	NS	NS	n/a	n/a	NS	n/a
Age	NS	F(6)=2.290, *P*=0.033	NS	NS	F(6)=4.182, *P*<0.001	F(6)=4.935, *P*<0.001
Marital status	F(3)=9.930, *P*<0.001	NS	NS	NS	NS	F(3)=9.228, *P*<0.001
Social class	NS	NS	NS	NS	NS	NS
Ethnic group	NS	NS	NS	NS	NS	F(3)=4.160, *P*=0.006

*Referral delay*						
Sex	F(1)=12.791, *P*<0.001	NS	n/a	n/a	F(1)=6.834, *P*=0.009	n/a
Age	F(6)=14.107, *P*<0.001	F(6)=3.904, *P*=0.001	F(6)=2.568, *P*=0.018	F(6)=3.791), *P*=0.001	F(6)=3.176, *P*=0.004	F(6)=32.482, *P*<0.001
Marital status	F(3)=5.435, *P*=0.001	NS	NS	NS	NS	F(3)=3.702, *P*=0.011
Social class	F(7)=2.230, *P*=0.029	NS	NS	NS	NS	NS
Ethnic group	F(3)=10.153, *P*<0.001	NS	NS	F(3)=5.186, *P*=0.001	NS	F(3)=6.102, *P*<0.001

*Secondary care delay*						
Sex	F(1)=23.119, *P*<0.001)	F(1)=4.854, *P*=0.028	n/a	n/a	NS	n/a
Age	F(6)=12.835, *P*<0.001	F(6)=3.025, *P*=0.006	NS	F(6)=68.596, *P*<0.001	F(6)=68.596, *P*<0.001	F(6)=88.649, *P*<0.001
Marital status	F(3)=11.713, *P*<0.001	NS	NS	F(3)=25.1921, *P*<0.001	F(3)=9.659, *P*<0.001	F(3)=34.609, *P*<0.001
Social class	F(7)=4.288, *P*<0.001	NS	F(6)=2.638), *P*=0.015	F(6)=2.553, *P*=0.013	NS	F(7)=9.150, *P*<0.001
Ethnic group	NS	F(3)=5.881, *P*=0.001	NS	NS	NS	NS

NS=not significant (*P*>0.05).

n/a=not applicable.

**Table 3:** Summary of significant findings from Generalised Linear Modelling (GLM)

	**Colorectal**	**Lung**	**Ovarian**	**Prostate**	**NHL**	**Breast**
*Total delay*						
Sex	NS	NS	n/a	n/a	NS	n/a
Age	F(1)=7.256, *P*=0.007	NS	NS	NS	F(1)=6.746, *P*=0.009	NS
Marital status	F(3)=6.672, *P*<0.001	NS	NS	NS	NS	F(3)=11.251, *P*<0.001
Social class	NS	NS	NS	F(7)=2.045, *P*=0.046	NS	NS
Ethnic group	NS	NS	NS	NS	NS	(F(7)=5.772, *P*=0.001

*Pre-hospital delay*						
Sex	NS	NS	n/a	n/a	NS	n/a
Age	NS	F(1)=5.688, *P*=0.017	NS	NS	F(1)=14.660, *P*<0.001	NS
Marital status	(F(3)=8.435, *P*<0.001	NS	NS	NS	NS	F(3)=11.593, *P*<0.001
Social class	NS	NS	NS	NS	NS	NS
Ethnic group	NS	NS	NS	NS	NS	F(3)=6.813, *P*<0.001

*Referral delay*						
Sex	F(1)=264.126, *P*<0.001	NS	n/a	n/a	NS	n/a
Age	F(1)=63.064, *P*<0.001	F(1)=15.682, *P*<0.001	NS	F(1)=12.933, *P*<0.001	F(1)=4.759, *P*=0.029	F(1)=129.733, *P*<0.001
Marital status	NS	NS	NS	NS	NS	F(3)=2.943, *P*=0.032
Social class						
Ethnic group	F(3)=3.213, *P*=0.022	NS	NS	NS	NS	NS

*Secondary care delay*						
Sex	F(1)=26.417, *P*<0.001	NS	n/a	n/a	F(1)=8.036, *P*=0.0005	n/a
Age	(F(1)=25.808, *P*<0.001)	NS	NS	F(1)=260.475, *P*<0.001	F(1)=34.651, *P*<0.001	F(1)=96.177, *P*<0.001
Marital status	F(3)=6.013, *P*<0.001	NS	NS	F(3)=4.731, *P*=0.0003	F(3)=3.780, *P*=0.010	F(3)=3.213, *P*=0.022
Social class	F(7)=2.247, *P*=0.028		F(6)=3.627, *P*=0.001	F(7)=2.357, *P*=0.021	NS	F(7)=3.602, *P*=0.001
Ethnic group	NS	F(3)=7.004, *P*<0.001	NS	NS	NS	NS

NS=not significant (*P*>0.05).

n/a=not applicable.
